# The Trojan Horse Liposome Technology for Nonviral Gene Transfer across the Blood-Brain Barrier

**DOI:** 10.1155/2011/296151

**Published:** 2011-11-16

**Authors:** Ruben J. Boado, William M. Pardridge

**Affiliations:** ^1^Department of Medicine, UCLA, Warren Hall 13-164, 900 Veteran Avenue, Los Angeles, CA 90024, USA; ^2^ArmaGen Technologies, Inc., Santa Monica, CA 90401, USA

## Abstract

The application of blood-borne gene therapy protocols to the brain is limited by the presence of the blood-brain barrier (BBB). Viruses have been extensively used as gene delivery systems. However, their efficacy in brain is limited by the lack of transport across the BBB following intravenous (IV) administration. Recent progress in the “Trojan Horse Liposome” (THL) technology applied to transvascular non-viral gene therapy of the brain presents a promising solution to the trans-vascular brain gene delivery problem. THLs are comprised of immunoliposomes carrying nonviral gene expression plasmids. The tissue target specificity of the THL is provided by peptidomimetic monoclonal antibody (MAb) component of the THL, which binds to specific endogenous receptors located on both the BBB and on brain cellular membranes, for example, insulin receptor and transferrin receptor. These MAbs mediate (a) receptor-mediated transcytosis of the THL complex through the BBB, (b) endocytosis into brain cells and (c) transport to the brain cell nuclear compartment. The expression of the transgene in brain may be restricted using tissue/cell specific gene promoters. This manuscript presents an overview on the THL transport technology applied to brain disorders, including lysosomal storage disorders and Parkinson's disease.

## 1. Introduction

DNA-based therapeutics may become a new generation of drugs for the treatment of brain disorders provided that the problem of its delivery across the blood-brain barrier (BBB) and into brain cells is solved. A global distribution of the transgene throughout the brain is needed for most of the enzyme replacement therapy protocols, and this could be possible by the transvascular route to brain via transport across the BBB. However, in the absence of either facilitated or receptor mediated transport systems, only lipophilic molecules of less than 400 Da are able to cross the BBB by simple diffusion [1]. Naked DNA molecules are not transported through this barrier [2–4]. Viruses have been used as brain DNA delivery systems with disappointing results associated with preexisting immunity, immunological response induced by viral coat proteins, and inflammation that led to demyelination [5–15]. Cationic lipids are widely used for transfection of DNA in in vitro tissue culture models. However, cationic lipid-DNA complexes in vivo are unstable or form large molecular weight aggregates that deposit in the pulmonary vascular bed [16–18], which decreases its bioavailability for delivery to the brain.

**Figure 1:**
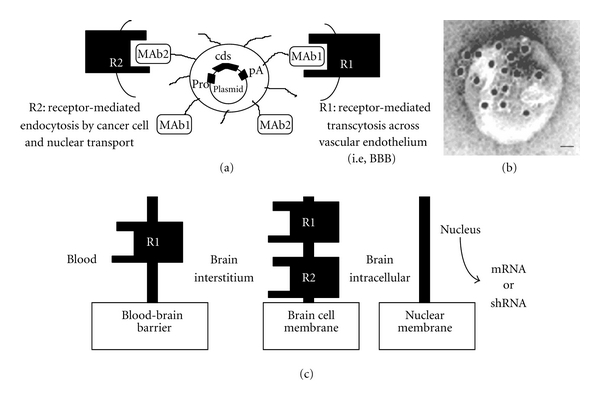
Engineering of Trojan horse liposomes (THL). (a) A supercoiled plasmid DNA is encapsulated in the interior of the THL. The plasmid encodes for a coding sequence (cds), the expression of which is under the influence of a promoter (pro), that is, SV40, and a polyadenylation sequence (pA). The surface of the liposome contains several thousand strands of 2000 Da polyethylene glycol (PEG) to stabilize the complex in blood. Approximately 1-2% of the PEG strands are conjugated with a targeting receptor- (R-) specific monoclonal antibody (MAb) (Table 1), which triggers transport of the THL across biological barriers in vivo. THLs are engineered with a single type of MAb to target both the BBB and brain cells in the same species. In an experimental mouse model of a human brain tumor, the THL is engineered with both the 8D3 mouse transferrin receptor (TfR) MAb (MAb1) to target the mouse BBB (i.e., R1) and the 8314 human IR MAb (MAb2) to target the human tumor cells (i.e., R2). Thus, the THL is transported through the mouse BBB via receptor-mediated transcytosis on the mouse TfR, and then through the intracranial human glioma cell membrane via endocytosis on the human insulin receptor. (b) Transmission electron microscopy of a THL. Mouse IgG molecules tethered to the tips of the PEG strands on the surface of the THL were detected with a conjugate of 10 nm gold and an antimouse secondary antibody. The position of the gold particles illustrates the relationship of the PEG-extended MAb and the liposome surface. Magnification bar = 20 nm. (c) The 3-barrier model for gene therapy of the brain. Following intravenous injection, the THL carrying the transgene must traverse 3 barriers in series to be able to reach the nucleus for expression: (a) the blood-brain barrier (BBB), (b) the brain cell membrane (BCM), and (c) the nuclear membrane. THLs can be engineered with a single type of MAb to target the same receptor in both the BBB and BCM (R1) or with 2 different MAbs to target different receptors at the BBB and the BCM, for example, R1 and R2, respectively. From [4].

An alternative approach for DNA delivery to the central nervous system (CNS) is the “Trojan horse liposome” (THL) technology [3, 4, 19–23] (Figure 1(a)). The construction of THLs has been optimized for plasmid DNA encapsulation [19]. The encapsulation of the transgene in the interior of a liposome protects the coding DNA against degradation by ubiquitous nucleases. Any DNA not fully encapsulated in the interior of the THL is removed by treatment of the THL with a mixture of exo/endonucleases. The THL is constructed with polyethylene glycol- (PEG-) conjugated lipids, and the PEG strands on the surface of the THL stabilizes the liposome in vivo and increases the plasma residence time [24, 25]. A small fraction of the PEG molecules, that is, 1-2%, carry a terminal maleimide functional group to allow for conjugation of the liposome surface with thiolated targeting ligands. The targeting ligand acts as a molecular Trojan horse (MTH) and is directed at an endogenous BBB receptor/transporter, such as the insulin receptor (IR) or transferrin receptor (TfR) receptor (Table 1) [3, 4, 19–23]. Widely used MTHs included peptidomimetic monoclonal antibodies (MAb) against BBB receptors. The extension of the PEG-conjugated MAb from the surface of the THL is illustrated by electron microscopy (Figure 1(b)). The IR or TfR are also expressed on the plasma membrane of brain cells, which enables the THL to traverse the brain cell membrane following delivery across the BBB (Figure 1(c)). MAbs against the IR or TfR are almost always species specific, and a MAb against the mouse TfR will not recognize the TfR on human cells. Therefore, in mixed animal models such as a brain tumor model produced by the intracranial growth of a human glioma in the mouse, a combination of targeting MAbs is used, so that the THL is targeted across both the mouse BBB and the human tumor cell membrane. For example, THLs were constructed with a MAb to the mouse TfR, to target the THL complex across the mouse BBB, and with a second MAb against the human insulin receptor (HIR), to target the THL across an intracranial human U87 glioma, as illustrated in Figure 1(a) [23]. With the development of genetically engineered forms of the HIRMAb, the THL technology may be translated to humans [26]. The engineering of plasmid DNA encoding the therapeutic transgene under the influence of brain cell-specific promoters eliminates ectopic transgene expression and enables transgene expression in targeted regions of the CNS [2, 19–23, 27, 28]. 

## 2. Trojan Horse Liposome (THL) Technology 

THLs are pegylated liposomes containing a supercoiled plasmid DNA molecule in the interior of the liposome (Figure 1(a)). THLs are engineered with a mixture of naturally occurring lipids that has been optimized for the encapsulation of plasmid DNA [4, 19]. The liposomes are comprised of 93% 1-palmitoyl-2-oleoyl-sn-glycerol-3-phosphocholine (POPC), 3% didodecyldimethylammonium bromide (DDAB), 3% distearoylphosphatidylethanolamine (DSPE)-PEG2000, and 1% DSPE-PEG2000-maleimide. The maleimide functional group allows for covalent conjugation of a thiolated MAb via a stable thioether linkage (Figure 1(a) and Table 1). A panel of species-specific peptidomimetic MAbs has been developed (Table 1), and their efficacy in delivering THLs to brain has been demonstrated in experimental animal models in vivo [1, 3, 4, 19–23, 27]. The 83-14 murine MAb to the HIR and the OX26 murine MAb to the rat TfR are used to target human and rat tissues, respectively (Table 1). The OX26 TfRMAb is active only in rats, so the rat 8D3 MAb against the mouse TfR is used in mice (Table 1) [20, 21, 27–33]. The 83-14 HIRMAb does not cross-react with the insulin receptor in rodents or even New World primates such as the squirrel monkey. However, this HIRMAb does cross-react with the insulin receptor of Old World primates such as the Rhesus monkey. Since the plasmid DNA must be delivered to the nuclear compartment of brain cells, the THL must traverse both the BBB and the brain cell plasma membrane (BCM) behind the BBB (Figure 1(c)). Owing to high expression of the TfR or IR on both the BBB and BCM barriers, the targeting MAb enables the sequential receptor-mediated transcytosis of the THL across the BBB followed by the receptor-mediated endocytosis of the THL into the brain cell (Figure 1(c)). THLs have also been successfully constructed to target human tumor cells in a scid mouse model wherein dual targeting MAbs were directed to the mouse TfR and HIR (Table 1 and Figure 1(a), i.e., MAb1 and MAb2) [23]. 

## 3. Brain Expression of Reporter Genes

In vivo applications of THLs were initially investigated with luciferase and lacZ reporter genes in vivo [3, 4, 20, 21, 34]. THLs were constructed with the expression plasmid of interest (i.e., luciferase reporter gene) and engineered with either the TfRMAb for rodents or the HIRMAb for Rhesus monkeys, respectively. The doses of DNA encapsulated in THLs and administered IV was 5 or 70 *μ*g per rat or primate, respectively, which are equivalent 20 and 12 *μ*g/kg body weight, respectively. When the transgene is driven by the widely read SV40 promoter, the levels of luciferase were *∼*10 pg luciferase/mg protein in the monkey brain. High levels of expression were also seen in peripheral tissues that are rich in the target receptor, including liver, spleen, and lung [27, 34]. A 50-fold increase in the tissue levels of luciferase was reported in primates, as compared to rat and mouse tissues. The high levels of expression in monkey tissues were associated with intrinsic properties of the HIRMAb that targets the nuclear compartment of the cell [4]. 

Time course studies in both rodents and primates demonstrated that the peak of luciferase expression occurs 48 hs following injection of a single IV dose of THLs. The levels of luciferase activity decline thereafter and as a function of time. There are 2 potential mechanisms for the decline in the expression of the transgene, that is, promoter inactivation and plasmid degradation. The levels of both luciferase enzyme activity and plasmid DNA decay in the primate brain and liver were measured, and both processes decayed with a *t*_1/2  _ of approximately 2 days, which indicates that the transient duration of the luciferase gene expression is mainly due to plasmid degradation [33].

**Figure 2:**
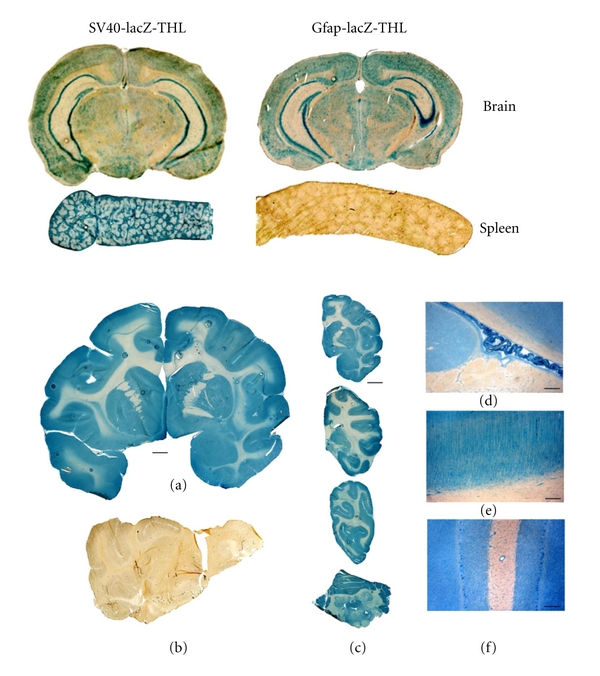
In vivo gene expression of a *β*-galactosidase reporter gene following systemic administration of THLs. (*Top panels*) *β*-galactoside histochemistry was performed on mouse brain and spleen removed 2 days after an IV injection of THLs carrying a *β*-galactosidase plasmid driven by either the SV40 promoter (SV40-lacZ-THL) (left panels) or Gfap promoter (Gfap-lacZ-THL) (right panels). THLs were targeted with the 8D3 antimouse TfRMAb. (*Bottom panels*) *β*-Galactosidase histochemistry of Rhesus monkey brain removed from either a monkey injected with THLs targeted with the HIRMAb ((a), (c), (d), (e), and (f)) or a control uninjected primate (b). The *β*-galactosidase expression plasmid is driven by the SV40 promoter. (a) shows a full coronal section of the primate forebrain. (c) shows half-coronal sections through the primate cerebrum and a full coronal section through the cerebellum; the sections from top to bottom are taken from the rostral to caudal parts of brain. (d, e, and f) are light micrographs of choroid plexus, occipital cortex, and cerebellum, respectively. All specimens are *β*-galactosidase histochemistry without counterstaining. The magnification in (a) and (b) is the same and the magnification bar in (a) is 3 mm; the magnification bar in (c) is 8 mm; the magnification bars in (d)–(f) are 155 *μ*m. Top panels are from [21]. Bottom panels are from [34].

The organ distribution of the lacZ transgene was also investigated at the cellular level with histochemistry following THL delivery of a reporter gene driven by the SV40 promoter and designated SV40-lacZ [4, 20, 34]. The latter was used to engineer THLs with either the TfRMAb or the HIRMAb (Table 1). The histochemical detection of the *β*-galactosidase is shown in Figure 2 for the mouse and the Rhesus monkey. The expression of the transgene was widely detected through the cortical and subcortical structures of mouse and monkey brain, with a greater gene expression in gray matter relative to white matter in both cerebrum and cerebellum (Figure 2). On the contrary, the *β*-galactosidase histochemistry of control uninjected primate brain shows no *β*-galactosidase activity (Figure 2(b)). Light micrographs of the primate brain shows gene expression within the choroid plexus epithelium (Figure 2(d)) and the capillary endothelium within white matter (Figure 2(f)). The gene expression was also confirmed within the neurons of the occipital cortex showing the columnar organization of this region in primate brain (Figure 2(e)). The molecular and granular layers of the cerebellum and the Purkinje cells were also positive for the transgene (Figure 2(f)). Confocal microscopy studies with antibodies against either bacterial *β*-galactoside or the neuronal neuN marker colocalized transgene expression in the neuronal compartment of brain [27, 34]. The ectopic expression of the *β*-galactosidase with the SV40-lacZ vector was also observed in tissues expressing either the TfR or the IR, such as spleen, providing that the transgene was driven by the widely read SV40 promoter (Figure 2, top left panel). 

It is possible to produce THLs that carry plasmid DNA engineered with a tissue-specific promoter [20]. This is of particular relevance in gene therapy protocols wherein ectopic expression of the transgene is not desired When the lacZ expression plasmid is driven by the brain-specific promoter derived from the 5′-flanking sequence of the glial fibrillary acid protein (Gfap) gene, the expression of *β*-galactosidase in brain was widely detected (Figure 2, top right panel), as previously seen with the SV40-lacZ plasmid (Figure 2, top left panel). On the contrary, there was no expression of the transgene in peripheral tissues (Figure 2, bottom right panel) when the transgene was under the influence of the brain-specific Gfap promoter. Tissue-specific gene expression with the combined use of THLs and the opsin promoter was demonstrated in vivo in the Rhesus monkey [35]. 

## 4. In Vivo Efficacy of THLs in a Model of Mucopolysaccharidosis

**Figure 3:**
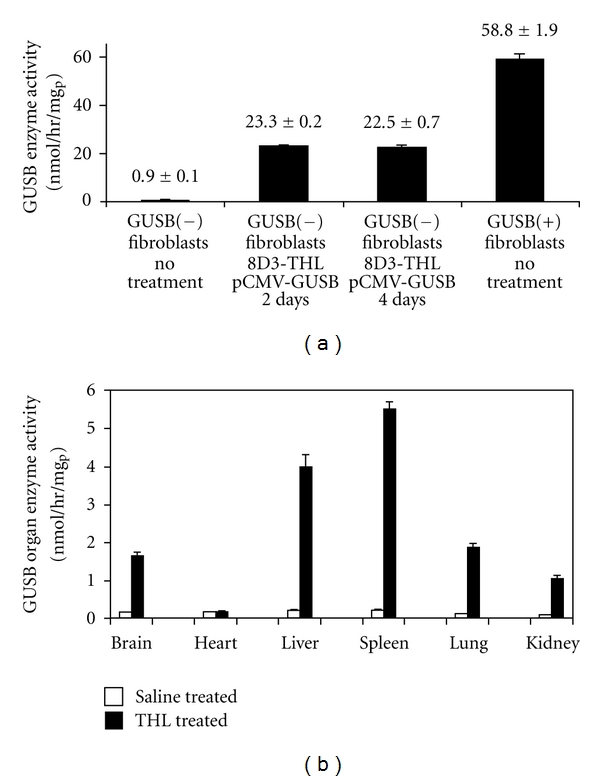
Enzyme replacement therapy with THLs in a mouse model of type VII mucopolysaccharidosis. (a) GUSB enzyme activity in GUSB null (−) fibroblasts and in fibroblasts obtained from wild type (+) mice. Fibroblasts were treated either with saline or with TfRMAb-targeted THLs encapsulated with the pCMV-GUSB expression plasmid. Data are mean ± SE (*n* = 4), and statistical significance was determined with ANOVA and Dunnett's test. The difference in GUSB enzyme activity in the THL-treated cells is significantly different from the untreated cells from the GUSB null mice (*P* < 0.01). (b) GUSB enzyme activity in brain and five other organs of GUSB null mice removed at 48 h after single intravenous administration of either saline or 10 ug/mouse of pCMV-GUSB plasmid DNA encapsulated in TfRMAb-targeted THLs. Mean ± SE (*n* = 4-5 mice/group). The difference in GUSB enzyme activity in the THL-treated mice, as compared to the saline-treated mice, is significant (*P* < 0.0005), in all organs, except the heart. From [36].

The in vivo efficacy of THLs was investigated in a model of type VII mucopolysaccharidosis (MPS), which is caused by mutations in the gene encoding the lysosomal enzyme *β*-glucuronidase (GUSB) [36]. MPS is a lysosomal storage disorder, and the majority of lysosomal storage disorders adversely affect the central nervous system [37]. Therefore, therapeutic transgenes must be delivered to all parts of the brain, and this is only possible with a transvascular route to brain. THLs were prepared with a plasmid DNA encoding for GUSB, and with the TfRMab to target THLs across both the BBB and the BCM in a transgenic mouse model of MPS-VII. The GUSB expression plasmid, designated pCMV-GUSB, is driven by the widely read cytomegalovirus (CMV) promoter. The latter was preferred over a brain-specific promoter, as MPS-VII affects both the CNS and peripheral tissues. The GUSB enzyme activity was investigated in cultured fibroblasts obtained from GUSB null mice [GUSB(−)], and >50-fold increase in the GUSB activity was observed following incubation with the THL carrying the pCMV-GUSB as compared with control GUSB(−) fibroblasts (Figure 3(a)). The GUSB enzyme activity persisted at high levels for over 2-week period [36].

In vivo studies in GUSB null mice were also performed with a single dose of 10 ug/mouse of pCMV-GUSB encapsulated in TfRMAb-targeted THLs. The GUSB enzyme activity in brain and peripheral organs was determined 48 hours after IV injection. GUSB enzyme activity was increased >10-fold in brain, liver, spleen, lung, and kidney, but not in heart (Figure 3(b)). The expression pattern of GUSB gene among mouse organs in vivo is consistent with the local expression of the TfR in the vascular barriers of these tissues. The liver and spleen are perfused with fenestrated capillaries that are highly porous, so the 100 nm THLs can freely cross their vascular barrier [20]. Heart, lung, and kidney are perfused with capillaries with continuous endothelial barriers [38]. Thus, the observation that GUSB enzyme activity is increased in lung and kidney with TfRMAb-targeted THLs in vivo provides additional evidence for the expression of the TfR on the vascular barrier in these organs in the mouse [27]. Lack of expression in heart supports prior work with reporter genes showing that TfRMAb-targeted THLs are not delivered across the vascular barrier in heart [20, 21, 27]. The brain GUSB enzyme activity observed at 48 h after a single THL administration approximated 2 U/mg protein (Figure 3(b)), which represents 55% of the brain level in heterozygotes [39]. Since the replacement of just 1–5% of lysosomal enzyme activity in an organ may be sufficient to cause therapeutic effects and a reversal of lysosomal storage disease [37], the levels of GUSB enzyme activity generated in the brain of null mice with a single IV injection of THLs is within the therapeutic range. The plasmid DNA is expressed episomally in brain cells without integration into the host genome [33]. Therefore, long-term treatment of lysosomal storage disorders with intravenous administration of THLs will require repeat administration of the gene medicine at intervals that are determined by both the persistence of transgene expression and the turnover of the expressed protein in brain and peripheral organs.

## 5. Brain Expression of Therapeutic Genes in a Model of Parkinson's Disease

The therapeutic efficacy of THLs was demonstrated in vivo in a model of Parkinson's disease (PD), wherein the therapeutic gene encoded for tyrosine hydroxylase (TH) [30]. PD is associated with a loss of dopaminergic neurons in the substantia nigra, which terminate in the striatum [40, 41]. The rate limiting enzyme in the synthesis of dopamine is TH, and a potential treatment for PD is TH gene replacement therapy. 

**Figure 4:**
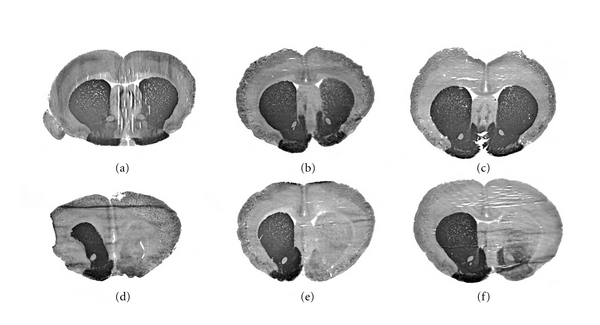
Levels of TH in brain following TH-gene therapy in the 6-OHDA Parkinson's disease model. The TH immunocytochemistry was performed in rat brains removed 72 hours after a single intravenous injection of 10 *μ*g per rat of clone 951 plasmid DNA encapsulated in THL targeted with either the TfRMAb (a, b, and c) or with the mouse IgG2a isotype control (d, e, and f). Coronal sections are shown for 3 different rats from each of the two treatment groups. The 6-hydroxydopamine was injected in the medial forebrain bundle of the right hemisphere, which corresponds to right side of the figure. Sections are not counterstained. The animals that received the TH gene therapy had a normalization of the brain TH levels as compared to the animals administered the nontargeted THLs, which showed complete lost of immunoreactive TH in the same region. From [30].

Studies were performed in the rat 6-hydroxydopamine (6-OHDA) model, and with THL packaged with a TH expression plasmid driven by the Gfap brain-specific promoter, designated clone 951 [30]. Gfap-TH-THLs were constructed with the OX26 MAb to target the rat TfR (Table 1). The intracerebral injection of 6-OHDA produced a 98% reduction in the levels of TH in the ipsilateral striatum as compared with the contralateral or nonlesioned control animals (Table 2). Animals with positive lesion were identified by apomorphine-induced contralateral rotation and administered 10 *μ*g clone 951 DNA encapsulated in either OX26-THL or in THLs targeted with a non-specific isotype control IgG1a mouse IgG, as a negative control [30]. The apomorphine-induced contralateral rotation test was used to demonstrate therapeutic efficacy. In the negative control group administered with the 951-THLs targeted with the non-specific IgG2a, the drug-induced rotation increased in all animals [30]. On the contrary, in the rats injected with the 951-THLs targeted with the TfRMAb, there was an 82% reduction in the apomorphine-induced contralateral rotations [30]. The therapeutic effect of the TH gene replacement was correlated with the levels of TH determined by enzyme activity (Table 2) or immunocytochemistry (Figure 4). The latter was performed in coronal sections of brain and showed complete normalization of the immunoreactive TH in the striatum of 6-OHDA lesioned rats 3 days after a single injection of the gene therapy (Figures 4(a)–4(c)). In contrast, lesioned control animals treated with the THLs targeted with the non-specific IgG2a isotype control antibody show a marked reduction in striatal immunoreactive TH (Figures 4(d)–4(f)). The levels of the TH enzyme activity were also normalized in the ipsilateral striatum (Table 2). Additional studies were performed in the 6-OHDA PD rat model with THLs carrying the TH gene under the widely read SV40 promoter, that is, clone 877 (Table 2) [22]. Similar data were obtained in both the restoration of the TH expression pattern in brain and in the reduction of the apomorphine-induced contralateral rotation [22]. The only difference between the studies performed with the TH expression plasmid driven by the SV40 promoter, or the Gfap promoter, was a 10-fold increase in the levels of TH activity in liver of animals injected with the SV40-TH construct, which is not seen with the Gfap-TH plasmid (Table 2 and Figure 2). The stability of the TH is associated with the availability of the biopterin cofactor, and the expression of the TH enzyme is found in regions of the brain that express GTP cyclohydrolase 1 (GTPCH) [42–44]. The GTPCH is also expressed in peripheral tissues, like liver [45], which supports the increased expression in liver TH activity when the TH transgene is driven by the SV40 promoter (Table 2) [22]. The gene therapy in this PD model with either SV40- or Gfap-TH plasmids produced normalization of the expression pattern of TH and without expression of supranormal levels of TH activity (Table 2) [22, 30]. This observation parallels findings observed in TH transgenic mice, which showed only a minor increase in either immunoreactive TH or TH activity in striatum despite a 50-fold increase in the level of TH mRNA in the substantia nigra [46]. The latter, in conjunction with the TH gene therapy with THLs (Table 2 and Figure 4), suggests that the expression of the TH gene is regulated at the posttranscriptional level in brain, so that the striatal TH activity is maintained within a narrow range [47] and by neurons expressing the GTPCH cofactor gene. 

**Figure 5:**
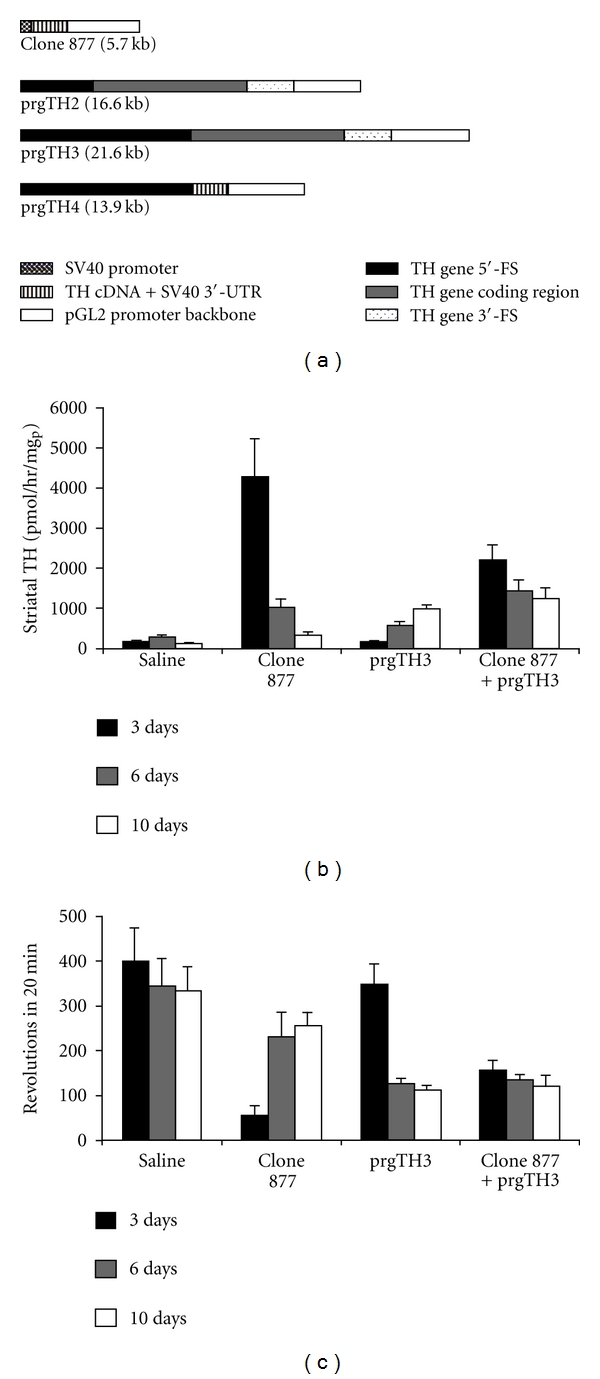
Enzyme replacement therapy in a Parkinson's disease model using THLs and TH genomic expression vectors. (a) Diagrams of four rat TH expression plasmids. The poly(A) transcription termination sequence is the SV40 3′-untranslated region (UTR) derived from the pGL2 promoter vector (Promega) for both clone 877 and prgTH4, whereas the poly(A) signal for prgTH2 and prgTH3 is derived from the rat TH gene. (b) Striatal TH enzyme activity on the side ipsilateral to the toxin lesion at 3, 6, and 10 days after a single injection of either saline or THLs carrying clone 877 alone, prgTH3 alone, or clone 877 + prgTH3 combined. Data are mean ± S.E. (*n* = 3–6 rats per point), and statistically significant differences were determined by Student's *t*-test. All plasmids were delivered to rat brain following the intravenous injection of TfRMAb-targeted THLs. The TH activity at 3 days following combination therapy is significantly greater than prgTH3 alone at 3 and 6 days after injection (*P* < 0.005) and is greater than clone 877 alone at 10 days after injection (*P* < 0.005). (c) Apomorphine-induced rotations at 3, 6, and 10 days after a single injection of either saline or THLs carrying clone 877 alone, prgTH3 alone, or clone 877 + prgTH3 combined. Data are mean ± S.E. (*n* = 3–6 rats per point). The rotation behavior at 3 days following combination therapy is significantly reduced as compared to prgTH3 alone at 3 days after injection (*P* < 0.005) and is significantly reduced as compared to clone 877 alone at 10 days after injection (*P* < 0.005). From [48].

As discussed above for the GUSB gene therapy, the plasmid DNA in THL is not integrated into the host genome [33]. Therefore, long-term treatments with repetitive intravenous administration of THLs are needed to produce a long-term therapeutic effect. The engineering of plasmid DNA vectors that incorporate intronic or chromosomal-derived gene elements may produce more sustained expression of the transgene following THL delivery. Therefore, a TH expression plasmid was engineered which incorporated the TH gene [48]. A series of 4 rat TH expression plasmids, designated clone 877, prgTH2, prgTH3, and prgTH4, were derived from the rat TH gene or cDNA, as outlined in Figure 5(a). Clone 877 is comprised of the TH cDNA driven by the SV40 promoter. Clone prgTH2 encodes a 12 kb TH genomic expression cassette, which includes a 3.0 kb TH 5′-flanking sequence (FS), the 7.3 kb rat TH coding region, and a 1.9 kb 3′-FS. The 3 kb rat TH 5′-FS in prgTH2 was expanded to 8.4 kb with the engineering of clone prgTH3. The introns and 3′-FS were eliminated by engineering clone prgTH4 (Figure 5(a)).

The cDNA form of TH gene therapy, clone 877, caused a 26-fold increase in striatal TH enzyme activity at 3 days after the IV injection, but this declined over 12-fold by 10 days (Table 3). There was a significant 86% improvement in motor function at 3 days after the injection of clone 877, but this improvement was not significant at 6 and 10 days after the single IV injection (Table 3). The genomic TH expression plasmids produced in general a lower peak of striatal TH enzyme activity in vivo, but a more lasting therapeutic effect. Striatal TH enzyme activity at 3 days after IV injection of prgTH2, or prgTH4, was less than that observed with clone 877, but the striatal TH enzyme activity at 10 days after injection with prgTH2 was significantly higher than with clone 877 (Table 3). The IV administration of prgTH3 resulted in no significant increase in striatal TH enzyme activity at 3 or 6 days after administration, relative to clone 877 or prgTH2, but yielded the highest striatal TH enzyme activity, and the lowest drug-induced rotation, of any single therapy at 10 days after administration (Table 3). Therefore, the clone 877 cDNA form of TH gene therapy is fast acting with short duration, whereas the genomic form of TH gene therapy with prgTH3 is slow acting with long duration (Table 3). The combination cDNA/genomic TH gene therapy was further investigated with THLs carrying both 877 and prgTH3 plasmids (Figures 5(b) and 5(c)). The striatal TH enzyme activity was significantly higher with the combination gene therapy as compared to clone 877 alone at 10 days after injection, and it was significantly higher as compared to prgTH3 alone at 3 and 6 days after injection (Figure 5(b)). The combination therapy also produced a parallel reduction in apomorphine rotation behavior (Figure 5(c)). The rotation behavior was significantly reduced with combination gene therapy as compared to clone 877 alone at 10 days after injection, and it was significantly reduced as compared to prgTH3 alone at 3 days after injection. 

In summary, combination gene therapy is superior to single cDNA gene therapy. The combination gene therapy using both short-acting cDNA-derived TH transgene and long-acting genomic-derived TH transgene provides a more sustained therapeutic duration in experimental PD as compared to single gene therapy using either cDNA-derived or genomic-derived transgene.

## 6. Long-Term Treatment with THL

Plasmid DNA-based gene therapy with THL technology involves episomal gene expression and must be given on a chronic basis, which raises concerns about potential toxic side effects from chronic repeat THL dosing. A 6-week toxicological study was conducted with repeated weekly intravenous administration of THLs carrying a 7 kb expression plasmid encoding for rat TH and targeted with either the OX26 MAb to the rat TfR or with the mouse IgG2a isotype control antibody [49]. Animals were divided into 3 treatment groups: (a) saline, (b) 5 ug DNA/week of the THLs targeted with the TfRMAb, and (c) 5 ug DNA/week of the THLs targeted with the nonspecific isotype control IgG2a antibody. At the end of 6 weeks of chronic weekly treatment, there was no measurable differences in the 3 groups with respect to body weights, 14 serum chemistries (Table 4), or organ histology of brain, liver, spleen, kidney, heart, or lung. The immunocytochemistry showed no evidence of inflammation in brain using antibodies that react with multiple components of the immune system [49]. These results demonstrate the lack of toxicity of chronic dosing of MAb-targeted THLs carrying plasmid DNA.

## 7. Formulation of THL

The efficiency of gene delivery to the brain and gene expression in target cells with THLs may be potentially enhanced by optimizing the formulation of THLs. The use of ethanol-mediated DNA condensation was recently shown to increase the efficiency of DNA encapsulation in THLs [50], and the polymer polyethylenimine (PEI) allowed for encapsulation of PEI/oligodeoxynucleotide polyplexes in THLs [51]. Avidin-biotin technology may also facilitate conjugation of ligands to THLs [52]. MAbs directed to the mouse or rat TfR and the human HIR are the most potent BBB Trojan horses developed to date for drug delivery across the mouse, rat, or primate BBB, respectively [26, 53–55], and the THL technology has been validated in numerous animals models (see above). As new targeting molecules with increased brain uptake, as compared to TfR- and HIR-MAb, become available, it may also be possible to engineer THLs with improved brain uptake and therapeutic efficacy. 

Other ligands have been tested in the construction of DNA liposomes, but demonstrated limitations in terms of specificity and/or global distribution of the transgene in the brain. Tat-peptide-modified liposomes were able to target human brain tumors in mice, but not the normal brain adjacent to the tumor [56]. Immunoliposomes labeled with anti-GFAP MAb targeted gliomas that had disruption of the BBB, but they were unable to penetrate unimpaired BBB [57]. Glycosylation of DNA lipoplexes and liposomes have been proposed to increase biodistribution most likely via absorptive endocytosis [58, 59]; however, the application of these constructs to gene delivery to the brain remains to be demonstrated.

## 8. Conclusions and Future Directions

The THL plasmid DNA gene transfer technology has been validated in multiple animal models in mice, rats, and Rhesus monkeys, and this work shows that it is possible to deliver transgenes to brain following the noninvasive intravenous administration of nonviral formulations. The ectopic expression of the transgene is shown to be eliminated by the combined use of THLs and plasmid DNA engineered with tissue-specific gene promoters. Transgene expression following THL delivery is reversible secondary to degradation of the plasmid DNA, which is not integrated into the host genome. This nonintegrating property of plasmid DNA is considered advantageous, since the integration of viral genomes into the host DNA can lead to insertional mutagenesis. Increase in the duration of plasmid DNA expression is possible with the engineering of plasmid DNA that incorporates chromosomal elements. THLs can be administered chronically without toxicity or immune reactions

The THL technology can be translated to humans with the use of human-specific antibodies that are genetically engineered to reduce immunogenicity. The murine HIRMAb, which is active at the human BBB, has been genetically engineered, and a humanized HIRMAb has been produced [26]. Therefore, it is possible to produce THLs with the humanized HIRMAb for gene transfer to the human brain (Table 1).

## Figures and Tables

**Table 1:** Targeting MAbs for THL and target tissue.

Targeting MAb	Target receptor	Experimental model and target tissue
Murine OX26 [53]	Rat TfR	(a) Rat C6 or RG2 glioma in culture (b) Rat C6-790 in cultures (c) In vivo transport via rat BBB and rat brain cells (neuron and glial). Gene delivery
Rat 8D3 [54]	Mouse TfR	In vivo transport via rat BBB and rat brain cells (neuron and glial). Gene delivery
Murine 8314 [60]	Human IR	(a) Human U87 glioma cultures (b) In vivo transport via primate/human BBB and brain cells (neuron and glial). Gene delivery
8D3 + 8314 [23]	Mouse TfR + human IR	(a) Experimental human brain tumors in scid mice (b) In vivo transport via mouse BBB and brain primate/human cells (neuron and glial). Gene delivery
Chimeric anti-TfR [55]	Mouse TfR	Gene delivery in mice
Humanized anti-IR [26]	Human IR	Gene delivery in humans

**Table 2:** Tyrosine hydroxylase (TH) in brain and peripheral organs in the rat 3 days after intravenous injection of gene therapy with TH expression plasmids driven by either the SV40 promoter (clone 877) or the Gfap promoter (clone 951), respectively.

Organs	Saline (pmol/hr/mg_p_)	TfRMAb-THL/877 (pmol/hr/mg_p_)	TfRMAb-THL/951 (pmol/hr/mg_p_)
Ipsilateral striatum	128 ± 27	5177 ± 446*	5536 ± 395*
Contralateral striatum	6445 ± 523	5832 ± 391	5713 ± 577
Ipsilateral cortex	176 ± 30	132 ± 16	184 ± 38
Contralateral cortex	150 ± 36	150 ± 24	135 ± 25
Heart	29 ± 3	45 ± 8	31 ± 3
Liver	13 ± 2	130 ± 28**	18 ± 6
Lung	42 ± 13	74 ± 22	30 ± 6
Kidney	24 ± 2	35 ± 5	31 ± 8

*P < 0.01 difference from saline group (ANOVA with Bonferroni correction; *n* = 4 rats per group). Rats were lesioned with intracerebral injections of 6-hydroxydopamine; 3 weeks after toxin injection, the rats were tested for apomorphine-induced rotation behavior; those rats testing positively to apomorphine were selected for gene therapy, which was administered intravenously 4 weeks after toxin administration; all animals were euthanized 3 days after gene administration. From [30].

**Table 3:** Tyrosine hydroxylase (TH) in brain and apomorphine-induced contralateral rotation after intravenous injection of gene therapy with TH expression plasmids.

Treatment (group)	Days after Rx	Apomorphine revolutions before Rx	Apomorphine revolutions after Rx	Striatal TH (pmol/h/mg_p_)
Saline	3	324 ± 69*	401 ± 72	162 ± 36
	6	232 ± 43	342 ± 62	288 ± 42
	10	228 ± 23	331 ± 55	120 ± 18

Clone 877	3	196 ± 24	56 ± 20^b^	4286 ± 918^b^
	6	231 ± 47	230 ± 55	1015 ± 213^a^
	10	219 ± 35	256 ± 27	335 ± 79^a^

prgTH2	*3*	251 ± 38	123 ± 31^b^	1343 ± 176^b,c^
	6	295 ± 55	119 ± 34^a^	1535 ± 324^b^
	10	344 ± 27	443 ± 70^c^	884 ± 7^b,d^

prgTH3	*3*	317 ± 23	234 ± 38^d^	167 ± 35^d^
	6	197 ± 11	127 ± 11^a^	545 ± 117^a^
	10	202 ± 31	111 ± 9^b,d^	985 ± 101^b,d^

prgTH4	3	211 ± 28	100 ± 9^b^	1219 ± 137^b,c^
	6	234 ± 37	113 ± 20^b^	1621 ± 196^b^
	10	3 ± 64	249 ± 64	500 ± 86^b^

None	NA	0	0	5068 ± 168

Rx: single IV injection of TfRMAb-targeted THLs encapsulating the respective TH expression plasmid. *Mean ± S.E. (*n* = 3–6 rats per point). Statistically significant differences were determined by Student's *t*-test.

Clone 877 is the TH expression plasmid driven by the SV40 promoter. For details in the engineering of TH genomic vectors, clones prgTH2–TH4, please see Figure 5.

Difference with saline: *P* < 0.05^a^; *P* < 0.005^b^.

Difference with clone 877: *P* < 0.05^c^; *P* < 0.005^d^.

NA: not applicable.

From [48].

**Table 4:** Summary of serum chemistry in long-term treatment with THLs.

Assay	Units	Saline	mIgG2a-PIL	OX26-PIL
Sodium	mM	143 ± 1	142 ± 1	140 ± 1
Potassium	mM	4.4 ± 0.1	4.6 ± 0.1	4.6 ± 0.2
Chloride	mM	100 ± 1	100 ± 1	100 ± 1
CO_2_	mM	29 ± 1	29 ± 1	27 ± 1
Glucose	mg/dL	168 ± 8	160 ± 6	163 ± 4
Creatinine	mg/dL	0.45 ± 0.03	0.40 ± 0.01	0.45 ± 0.02
Urea nitrogen	mg/dL	19 ± 1	21 ± 2	18 ± 1
Total protein	g/dL	5.2 ± 0.1	5.3 ± 0.1	5.3 ± 0.1
Albumin	g/dL	1.4 ± 0.1	1.4 ± 0.1	1.4 ± 0.1
Bilirubin, total	mg/dL	0.35 ± 0.03	0.25 ± 0.05	0.33 ± 0.02
Alk phos	U/L	231 ± 27	212 ± 25	281 ± 11
AST (SGOT)	U/L	65 ± 5	59 ± 2	74 ± 6
ALT (SGPT)	U/L	54 ± 2	52 ± 3	59 ± 1
Calcium	mg/dL	9.4 ± 0.1	9.5 ± 0.2	9.2 ± 0.1

Data are mean ± SE (*n* = 6 rats in each of the three treatment groups). From [49].
